# Current Insights to Regulation and Role of Telomerase in Human Diseases

**DOI:** 10.3390/antiox6010017

**Published:** 2017-02-28

**Authors:** Mert Burak Ozturk, Yinghui Li, Vinay Tergaonkar

**Affiliations:** 1 Division of Cancer Genetics and Therapeutics, Laboratory of NFκB Signaling, Institute of Molecular and Cell Biology (IMCB), A*STAR (Agency for Science, Technology and Research), Singapore 138673, Singapore; liyh@imcb.a-star.edu.sg (Y.L.); vinayt@imcb.a-star.edu.sg (V.T.); 2 Department of Biochemistry, Yong Loo Lin School of Medicine, National University of Singapore (NUS), Singapore 117597, Singapore; 3 Centre for Cancer Biology, University of South Australia and SA Pathology, Adelaide SA 5000, Australia

**Keywords:** telomerase, telomerase reverse transcriptase (TERT), hTERT promoter mutations, telomerase RNA component (TERC), telomere, cancer, age-related diseases

## Abstract

The telomerase ribonucleoprotein complex has a pivotal role in regulating the proliferation and senescence of normal somatic cells as well as cancer cells. This complex is comprised mainly of telomerase reverse transcriptase (TERT), telomerase RNA component (TERC) and other associated proteins that function to elongate telomeres localized at the end of the chromosomes. While reactivation of telomerase is a major hallmark of most cancers, together with the synergistic activation of other oncogenic signals, deficiency in telomerase and telomeric proteins might lead to aging and senescence-associated disorders. Therefore, it is critically important to understand the canonical as well as non-canonical functions of telomerase through TERT to develop a therapeutic strategy against telomerase-related diseases. In this review, we shed light on the regulation and function of telomerase, and current therapeutic strategies against telomerase in cancer and age-related diseases.

## 1. Introduction

Telomeres are short, non-protein coding hexanucleotide repeats of TTAGGG which localize at the end of the chromosomes in association with protective proteins collectively termed shelterin. The shelterin complex comprising Telomere Repeat Factor 1 (TRF1), Telomere Repeat Factor 2 (TRF2), Repressor/Activator Protein 1 (RAP1), Protection of Telomere 1 (POT1), Tripeptidyl-peptidase 1 (TPP1), TRF1- and TRF2-Interacting Nuclear Protein 2 (TIN2) provides protection against DNA damage signals, DNA recombination or DNA end-joining processes which may trigger chromosomal instability [1]. In somatic cells, telomeres shorten after each cell division [2], because the gap between the final RNA primer and end of the chromosome cannot be completed in the strand where replication is performed by Okazaki fragments, and 3’ overhangs occur [3]. This progressive telomere shortening following each cycle of cell division is commonly known as the “end replication problem” and when telomeres become critically short, cells undergo a senescent state termed “Hayflick limit”. They can live for years without division in this state [4], before encountering another barrier called “crisis” where a vast majority of cells undergo cell death. Pre-malignant cells can pass this barrier by regulating telomere length and they can become tumorigenic by acquiring oncogenic changes such as lack of function of cell cycle checkpoints as well as tumor suppressors. The length of telomeres is majorly regulated by the activation of telomerase or, in rare cases, by alternative lengthening of telomeres (ALT) mechanisms involving homologous recombination [5]. Since the dominant regulation mechanism of telomeres is dependent on the telomerase activity, we focus on telomerase in this review.

Telomerase is a ribonucleoprotein enzyme, which is responsible for lengthening of telomeric repeats at the end of chromosomes. This multi-subunit complex consists of the telomerase reverse transcriptase (TERT) catalytic subunit, telomerase RNA component (TERC) which is the RNA template used for the synthesis of telomeres, dyskerin (DKC1), Nop10 [6], NHP2, Pontin and Reptin [7,8] as well as small ribonucleoproteins such as NHP2, NOP10, and GAR1 [9]. Assembly of telomerase occurs in the Cajal bodies and the trafficking of telomerase to telomeres are mediated by TCAB1 and TPP1 proteins [10].

## 2. Regulation and Role of Human TERT in Cancer and Aging

Normal human somatic cells and benign tissues have very low or undetectable telomerase activity and thus display a limited lifespan due to transcriptional silencing of *TERT* during differentiation [11]. In contrast, telomerase activity is observed in more than 80%–90% of tumors as well as in stem cells and adult germline tissues [11,12]. This observation supports the hypothesis that cancer cells may have originated from cancer stem cells [13], although there is also evidence supporting the notion that cancers can arise from de-differentiated progenitor cells or somatic cells that acquire stem-like properties [14]. Telomerase reactivation during carcinogenesis enables neoplastic cells to proliferate *ad libitum*, thus attaining a series of oncogenic genetic alterations necessary for their malignant development. On the other hand, although normal stem cells possess telomerase activity, they lose telomerase expression with increasing age, resulting in progressive telomere loss, stem cell dysfunction and aging syndromes [15]. Telomere shortening in normal somatic and stem cells is one of the major hallmarks of aging and a critical determinant of longevity [16]. This is primarily regulated by the expression of telomerase as the restoration of telomerase activity has been demonstrated to be sufficient in reverting age-related phenotypes in vivo [17]. Hence, telomerase plays a major role not only in cancer, but also in age-related diseases whose pathogenesis is dependent on its regulation of telomere length. 

TERT is the catalytic subunit of telomerase holoenzyme, which is essential for the extension of telomeres using TERC as a template. *TERT* gene expression is crucial for the reconstitution of telomerase activity and during carcinogenesis; telomerase is reactivated through the transcriptional de-repression of *TERT* in neoplastic cells [18,19]. Apart from cancer, impairment in TERT may cause telomere shortening, resulting in age-related diseases such as idiopathic pulmonary fibrosis (IPF), dyskeratosis congenita and aplastic anemia [20]. Therefore, it is an important therapeutic target in cancer.

The length of the human *TERT* (*hTERT*) gene located on chromosome 5 is approximately 42 kb and consists of 16 exons [21]. While 22 isoforms are expressed from this gene, only the full-length isoform which retains reverse transcriptase activity is able to directly elongate telomeres [22,23]. It has been reported that the *TERT* gene has alternative splice variants that lack catalytic activity, and these variants affect endogenous telomerase activity adversely through competitive inhibition, hence resulting in the modulation of telomere length [24]. Although h*TERT* splice variants are known to impair endogenous telomerase function through competitive binding of TERC, one variant—the β-deletion variant which lacks the reverse transcriptase domain—has been shown to mediate resistance to drug-induced apoptosis in a telomere-independent manner in cancer cells [25]. This suggests that the well-known feature of telomerase in promoting resistance to chemotherapeutics is independent of its catalytic activity. 

Human TERT (127 kDa) functions as a dimer in the telomerase complex [6], unlike ciliates [26,27]. The human TERT protein consists of four principal regions: N-DAT (N-terminal), TRBD (telomerase RNA binding domain), RT (reverse transcriptase domain), and C-DAT (C-terminal) including 14-3-3 binding site [28]. The reverse transcriptase (RT) motif is critical for adding repeats through interaction with telomerase RNA (TERC). Mutations in the reverse transcriptase motif can lead to the inhibition of telomerase activity and consequent telomere shortening [29]. hTERT can interact with proteins such as chaperone p23, HSP90, TEP1, Ku, hEST1, KIP, PinX1, which regulate post-translational modifications, telomerase assembly, localization and activity [30]. The interaction of 14-3-3, which is a chaperone-like protein [31], with hTERT, for example, regulates TERT intracellular localization and enhances its nuclear localization [32]. Mutations in the RNA binding domain (TRBD) may also impair the enzyme activity [33]. 

The *TERT* promoter has multiple binding sites for various transcription factors such as E-twenty six (ETS) family members, SP1, E2F, AP1, p53, HIF1, p21, C-MYC, NF-kB, β-Catenin [34,35,36,37,38,39,40,41,42,43,44]. In addition, hormone receptor-mediated signaling pathways such as estrogen receptor (ER)-signaling can regulate *TERT* expression [45]. While some of these transcription factors can regulate TERT expression in cancers, there is a distinct subset of cancers that display recurrent mutations in the TERT promoter, resulting in the binding of new transcription regulators. Recurrent somatic and germline mutations in the TERT promoter were first identified in sporadic and familial melanoma respectively. Both somatic and germline TERT mutations have been found to result in the de novo generation of binding motifs for the ETS family of transcription factors. In contrast to germline mutations, somatic mutations in the TERT promoter occur more frequently and have been identified in several cancer types besides human melanoma, including glioblastoma, hepatocellular carcinomas, bladder cancers, thyroid cancers, and urothelial cancers [46]. The high frequency of TERT promoter mutations in a multitude of advanced cancers implicates their role as a key mechanism of telomerase reactivation. These mutations commonly occur at positions −124 and −146 nucleotides from ATG, and create de novo binding sites for transcription factors including ETS1/2, GABP, p52 (NFkB2), resulting in upregulated *TERT* expression [47,48,49,50,51,52,53]. Acquisition of either of the *TERT* promoter mutations leads to the enhanced enrichment of active histone marks associated with an open chromatin state and the increased recruitment of Pol II (RNA polymerase II) on the mutant allele, resulting in upregulation of *TERT* expression [54]. These epigenetic changes induced by *TERT* promoter mutations support the observations by Chiba et al., which demonstrated that introduction of a *TERT* promoter mutation in stem cells is sufficient to block the epigenetic silencing of *TERT* expression during differentiation [55].

Recently, Akincilar et al. demonstrated that the de novo binding site for the alpha subunit of GA-binding protein (GABPA), an ETS transcription factor, at the mutant *TERT* promoter facilitates a long-range chromatin interaction with another GABPA at a distal enhancer to mediate TERT reactivation in cancer cells [56]. Besides long-range enhancer-promoter looping mechanisms, oncogenic signaling pathways such as the RAS-ERK pathway can regulate *TERT* reactivation at mutant *TERT* promoters in cancers harboring BRAF mutations [57]. Although MAPK (mitogen-activated protein kinase) and other mitogenic signaling pathways have been previously shown to regulate TERT expression at the wild-type promoter [58], Li et al. further demonstrated that ERK2 (extracellular signal–regulated kinases) can bind specifically to mutant TERT promoters to modulate its active chromatin state, thereby suggesting an additional regulatory mechanism by MAPK signaling at the TERT promoter in the presence of mutation [57]. Furthermore, common single-nucleotide polymorphisms (SNPs) such as rs2853669 found at the *TERT* promoter have been shown to modify the survival and prognosis of certain cancers carrying *TERT* promoter mutations [59], such as clear cell renal cell carcinoma, melanoma and glioblastoma, where patients exhibit improved survival in the presence of this polymorphism [60,61,62], plausibly through disruption of an adjacent ETS2 binding site [42,63]. However, there is also another study reporting the lack of impact on survival and metastasis by rs2853669 alone, whereas patients with rs2853669 and TERT promoter mutation together have exhibited poor prognosis [64]. This is suggesting that the effect of SNPs in modifying patient outcome may be dependent on the TERT promoter mutations.

While *TERT* is reactivated in the broad range of cancers, not all cancers possess mutations in the *TERT* promoter, suggesting that other mechanisms such as chromosomal rearrangements may play a role in *TERT* activation. For example, a chromosomal rearrangement resulting in translocation of the *IRX2* promoter to the *TERT* locus has been found to drive *TERT* expression in clear cell sarcoma of kidney [65]. In addition, rearrangements at the *TERT* promoter which appose super-enhancers to the *TERT* gene have been found to mediate *TERT* reactivation in high-risk neuroblastoma [66,67]. Moreover, *TERT* gene amplification can also cause higher *TERT* expression and telomerase activity [68,69].

## 3. Non-Canonical Function of Telomerase in Cancer and Aging

Apart from the canonical role of telomerase in cancers, telomere-independent functions of telomerase have been documented in animal studies. TERT upregulation is observed in certain murine cancers such as breast, skin [70,71] while the ectopic expression of TERT has been found to stimulate cell proliferation [72,73,74]. Studies using mammalian cells have shown that in immortalized cells that possess ALT mechanisms to maintain telomeres, overexpression of H-Ras could not transform cells. However, when TERT was co-expressed with H-Ras, these cells became tumorigenic and this transformation was independent of the catalytic activity of TERT, suggesting that hTERT has additional roles in cancer promotion that are distinct from its activity at telomeres [75]. Although TERT overexpression alone has been found to be insufficient to drive tumorigenesis unless cells lose the function of tumor suppressor genes such as *TP53*, *PTEN*, *RB* [76], various studies have shown that it promotes cancer progression in the presence of other oncogenic factors. One example is a study by Shang Li et al., which showed that silencing of TERT reduced cancer cell proliferation and growth without affecting telomere length [77]. Other supporting studies have shown that over-expression of hTERT in mammary epithelial cells reduced their dependence on external mitogens through regulation of proliferation genes [78], while the inhibition of telomerase induced apoptosis in ovarian cancer cells [79]. 

In addition, alternatively spliced variants of TERT which do not have telomerase activity are able to trigger cell proliferation and induce apoptosis inhibition mechanisms [25,78,80,81]. TERT can also interact with oncogenic transcription factors such as NF-κB subunit p65 (RelA) and this interaction has been found to mediate translocation of TERT into the nucleus in multiple myeloma cells [82,83]. In a more recent study, the interaction of TERT and p65 was found to be crucial for regulating the expression of NF-κB target genes which are essential for inflammation and cancer development [84]. Apart from the role of TERT in NF-κB-mediated transcription, TERT can also regulate the Wnt/β-Catenin signaling pathway. This link was first demonstrated in hair follicle stem cells, where the overexpression of catalytically inactive TERT stimulated the proliferative phase in keratinocytes through activation of Wnt pathway, thus resulting in the promotion of hair growth [85]. The interaction of TERT with Wnt/β-Catenin signaling was further confirmed by Park et al., who showed that expression of either wild-type TERT or catalytically inactive TERT led to the activation of Wnt reporters. Further experiments revealed that TERT and β-Catenin co-associated at the promoters of Wnt/β-Catenin target genes by forming a complex with chromatin remodeler BRG1 (also known as SMARCA4) [86]. TERT can also bind the promoter of Cyclin D1 and regulate cell-cycle progression in laryngeal squamous carcinoma [87]. The Wnt/β-Catenin pathway has been shown in turn to regulate the expression of TERT in embryonic stem cells as well as cancer cells through the recruitment of β-Catenin to *TERT* promoter, suggesting that a positive feedforward loop between telomerase and β-Catenin may exist to drive the progression of Wnt-dependent cancers [88]. Recently, TERT has also been shown to increase the expression of tRNAs through its association with RNA Pol III, which promotes the stabilization of Pol III at target promoters [89]. This induction of tRNA expression was found to drive the proliferation of cancer cells. This finding is important when targeting tRNAs in the cancer therapy, because TERT may be regulating a distinct pool of tRNAs that drive the proliferation of cancer cells. This is supported by the observation that the tRNA pool profile is different in normal differentiating cells compared with proliferative cells such as cancer cells. In normal cells, tRNAs translating mRNAs related to proliferation genes are suppressed as a way of blocking the onset of cancer [90]. When tRNAs associated with expression of proliferation genes are activated, they deregulate protein expression profile and cause the transformation of cells. In a further state, they can accelerate metastasis [91]. Moreover, recently it has been demonstrated that TERT augments cell adhesion and migration upon ectopic expression of TERT in ALT positive cells [92].

The role of TERT is not limited to the nucleus; it is also localized in the mitochondria. This finding strengthens the claim about a telomere-independent function of telomerase, because the vast majority of findings about telomerase and mitochondria are based on the regulation of cellular stress. It has been shown that TERT translocates to the mitochondria from the nucleus during oxidative stress to mediate its protective effects in non-tumor cells [93]. Activation of the mitochondria localization of TERT is triggered by the phosphorylation of TERT on tyrosine 707 by Src kinase. However, antioxidants such as N-acetyl cysteine can block the nuclear export of TERT [94]. Ahmed et al. showed that during oxidative stress, hTERT overexpression protected fibroblasts from H_2_O_2_ and etoposide-induced apoptosis. On the contrary, silencing hTERT increased reactive oxygen species (ROS) production in HUVEC [93]. It was found that cytosolic acidification increased after H_2_O_2_ treatment in HeLa cells. Normally, cytosolic acidification triggers apoptotic mechanisms such as translocation of Bax in mitochondria and cytochrome c release from mitochondria. However, overexpression of hTERT blocked the intrinsic apoptotic mechanism, leading to increased cell proliferation and colony formation [95]. In another study, overexpression of hTERT with a defect in the nuclear export signal (NES) prevented the migration of TERT from nucleus to mitochondria in fibroblasts, resulting in increased oxidative stress, mitochondrial and nuclear DNA damage, as compared to cells having a non-defect wild type TERT. This oxidative stress-mediated cell-cycle arrest could be rescued by treatment of antioxidant N-acetyl-cysteine (NAC) [96].

## 4. hTERC, an RNA Subunit of Telomerase

TERC, having a molecular weight of 153 kDa as a dimer, is a 451-nucleotide-long non-coding RNA (lncRNA) and acts as a template for telomerase to synthesize tandem telomeric repeats. It has been shown that interaction of TERC with TERT is sufficient for telomere synthesis in vitro [97]. Although the levels of TERT are limiting in somatic cells, TERC is abundant [98]. Recently, it has been shown that Poly(A)-specific ribonuclease that is cap-dependent poly(A) deadenylase encoded by *PARN* is necessary for maturation of TERC [99]. The half-life of TERC is long, depending on the presence of endogenous TERT. This can range from 3 to 4 weeks in cancer and stem cells [100]. TERC includes two conserved parts: CR2/CR3, which is the catalytically essential pseudoknot-template core part and CR4/CR5, which is a stem-loop part [101]. The CR4/CR5 part interacts with the RNA-binding domain of TERT, and this interaction is necessary for telomerase activity in vivo [102]. Apart from the role of TERC in regulation of telomerase activity, it has been reported that silencing of TERC affected angiogenesis and metastasis-related genes’ expression without affecting telomere length [103] and mutations in regulators of TERC such as PARN caused telomere attrition playing a role in aging.

## 5. Other Subunits of Telomerase

Dyskerin (DKC) has a molecular weight of 58 kDa and Nop10 is a 7.7 kDa small subunit. Although these subunits are found in the telomerase holoenzyme, they are not essential for telomerase activity in vitro [104,105]. However, DKC has been shown to be necessary for telomerase activity in vivo, through promoting the stabilization of TERC [106]. In addition, DKC plays a role in the biogenesis of p53 as well as the function of ribosomes [107]. The other telomerase subunits, Reptin and Pontin, can also play a role in chromatin remodeling as well as DNA damage repair [108]. These minor subunits are mainly known to regulate telomerase activity via interaction with TERC, which mediates the stability and assembly of telomerase in vivo [30].

## 6. Therapeutic Aspects of Telomerase-Targeted Treatment in Cancer and Aging

Telomeres are the molecular counters of cell division. In adult somatic cells, they shorten after each cell division due to insufficient telomerase activity. Telomere shortening is one of the major factors of aging [109]. However, early telomere shortening due to mutations in either telomerase or telomere-related genes like shelterin may cause premature age-related diseases called “telomeropathies” or “telomere syndromes”, such as pulmonary fibrosis, dyskeratosis congenita and aplastic anemia [110,111]. Apart from mutations, lifestyles that involve consuming unhealthy foods, smoking and obesity might lead to telomere shortening [112]. 

TERT overexpression represents a potential strategy to treat premature age-related diseases. However, this approach runs the risk of causing other diseases such as cancer. Constitutive expression of TERT in engineered mice which have higher expression of p53, p21 and p19ARF resulted in longer telomere length and delayed onset of age-related pathologies [113]. In another study, transient expression of TERT using non-integrative adeno-associated viral vector increased the lifespan and delayed the onset of age-related diseases including cancer [114]. Moreover, some compounds such as TA-65 have been shown to activate telomerase through c-Myc [115]. Hence, while devising a therapeutic strategy to activate *TERT* in patients who have age-related diseases, characterization of the strategy should be performed carefully to ensure that it does not affect mitogenic pathways or induce cancer development in patients with deficiency in tumor suppressor mechanisms.

**Figure 1:**
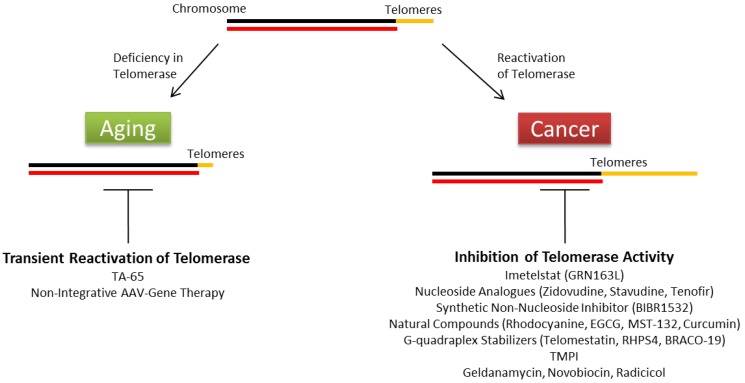
Summary of therapeutic strategies targeting telomerase activity in age-related diseases and cancer.

On the other hand, telomerase activity can be targeted to inhibit the development of cancers that have high activity. Telomerase is an attractive target of cancer therapy since most cancer cells have high telomerase activity while normal somatic cells have very low or undetectable activity. However, no suitable drug that targets telomerase has been successfully developed in the market due to the adverse effects on normal stem cells, whose functions depend on telomerase activity [116]. Currently, only one telomerase inhibitor, imetelstat (GRN163L), has progressed to clinical trials [117]. Imetelstat impairs the telomerase activity and telomere elongation by coupling with TERC, causing stable duplexes [118,119]. There are other groups targeting telomerase activity through different mechanisms, such as nucleoside analogues (zidovudine, stavudine, tenofovir), synthetic non-nucleoside inhibitors (BIBR1532), natural compounds (rhodocyanine, EGCG, MST-132, curcumin), G-quadraplex stabilizers (telomestatin, RHPS4, BRACO-19), isothiazolone derivates (TMPI) and HSP90 inhibitors (geldanamycin, novobiocin, radicicol) (reviewed in 91, summarized in Figure 1). However, these telomerase inhibitors generally exhibited non-specificity and cytotoxicity.

It has been reported that TERT protects cells from stress-induced DNA damage [93]. Therefore, this might be one of the reasons why radiotherapy or DNA damage-targeted chemotherapy does not usually lead to the complete eradication of cancer cells. Combination of those therapeutic strategies and inhibition of TERT expression apart from telomerase may help to overcome the recurrence of cancers. However, finding a good candidate that causes depletion of TERT in these cells is a major challenge considering previously failed candidates. Targeting telomerase reactivation mechanisms at mutant *TERT* promoters may be a more effective and promising approach to inhibit telomerase specifically in cancer cells while avoiding the cytotoxic effects to normal stem cells that do not contain *TERT* promoter mutations. Hence, more biochemical characterization of the complex and multi-faceted mechanisms of mutant *TERT* promoter activation in different cancers is necessary at this early stage.

## 7. Conclusions

Telomerase reactivation is seen in more than 80%–90% of tumors. On the contrary, deficiency in telomerase activity results in aging-related diseases. There are many factors regulating telomerase activity and its subunits. TERT, the catalytic subunit of telomerase, is the most important target among other telomerase subunits, because it has a major role in the reconstitution of telomerase activity as well as having various non-telomeric functions in cancer progression. Unfortunately, there is no effective drug targeting TERT and telomerase activity in the market to date. Disrupting the transcriptional activation of *TERT* at mutant *TERT* promoters represents a promising therapeutic strategy for the treatment of a subset of cancers with mutant *TERT* promoters. Transient expression of telomerase seems promising against premature age-related diseases. For cells that do not have a mutation in the *TERT* promoter in cancer cases or deficiency in the telomerase activity in age-related diseases, more studies should be performed to search for novel regulators and to characterize them.
